# Supplementary material for: Identification of photoperiod-induced specific miRNAs in the adrenal glands of Sunite sheep (Ovis aries)
Source: Front Vet Sci. 2022 Jul 22;9:888207. doi: 10.3389/fvets.2022.888207 (PMC9354845; doi:10.3389/fvets.2022.888207)
Supplement: Supplementary file 1 [file Data_Sheet_1.ZIP › Supplementary file/miRDeep2 file.zip!/miRDeep2 file/Novel_Structure/LP42A2/Novel_100.pdf]

Provisional ID	:	NC_019481.2_35117
Score total	:	123.4
Score for star read(s)	:	3.9
Score for read counts	:	115.9
Score for mfe	:	2.1
Score for randfold	:	1.6
Score for cons. seed	:	
Total read count	:	239
Mature read count	:	237
Loop read count	:	0
Star read count	:	2

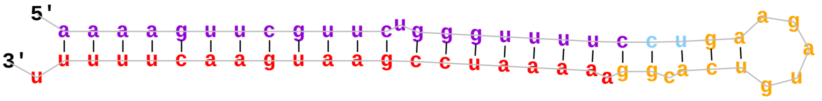

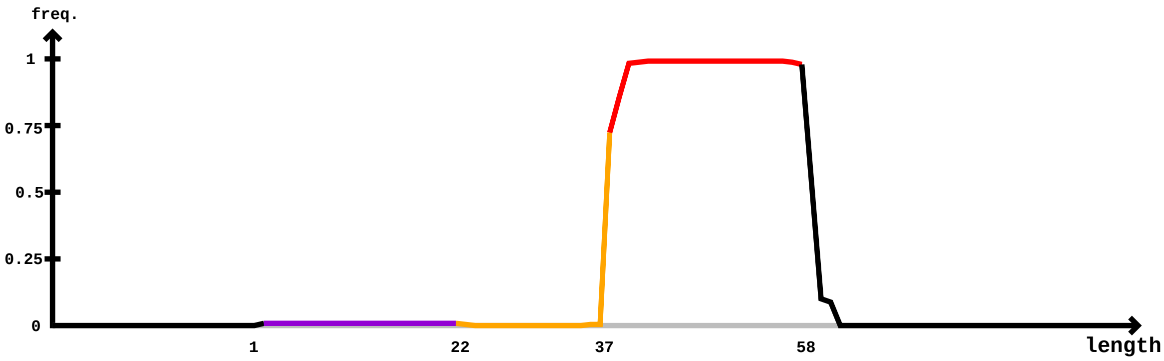

Star Mature

```
5'- gucuguuauuggguuggcccaaaaguucguucuggguuuuccugaagaugucacggaaaaauccgaaugaacuuuuuggucaccccaauacagucuuugccgacccucccu   -3'    obs
    gucuguuauuggguuggcccaaaaguucguucuggguuuuccugaagaugucacggaaaaauccgaaugaacuuuuuggucaccccaauacagucuuugccgacccucccu          exp
    (.((((.((((((.(((((.((((((((((((.((((((((((((.....))).)).).))))))))))))))))))).))))).)))))))))).)................   reads   mm   sample
    ....................aaaaguucguucuggUuuuuc......................................................................   1       1    seq
    ....................aaaaguucguucuggguuuuc......................................................................   1       0    seq
    ......................................................ggaaaaaCccgaaugaacuuu....................................   1       1    seq
    ........................................................aaaaaCccgaaugaacuuuu...................................   2       1    seq
    ........................................................aaaaaucUgaaugaacuuuuu..................................   111     1    seq
    ........................................................aaaaaucUgaaugaacuuuuug.................................   59      1    seq
    .........................................................aaaaCccgaaugaacuuuuu..................................   1       1    seq
    .........................................................aaaauccgaaugaacuuuuu..................................   8       0    seq
    .........................................................aaaaucAgaaugaacuuuuug.................................   1       1    seq
    .........................................................aaaaCccgaaugaacuuuuugg................................   2       1    seq
    .........................................................aaaauccgaaugaacuuuuugg................................   1       0    seq
    .........................................................aaaaucUgaaugaacuuuuuggu...............................   18      1    seq
    .........................................................aaaauccgaaugaacuuuuuggu...............................   1       0    seq
    ..........................................................aaaCccgaaugaacuuuuu..................................   28      1    seq
    ..........................................................aaauccgaaugaacuuuuu..................................   1       0    seq
    ..........................................................aaauccgaaugaacuuuuug.................................   1       0    seq
    ...........................................................aaCccgaaugaacuuuuuggu...............................   1       1    seq
    ............................................................aCccgaaugaacuuuuuggu...............................   1       1    seq
```
